# MFN1-dependent alteration of mitochondrial dynamics drives hepatocellular carcinoma metastasis by glucose metabolic reprogramming

**DOI:** 10.1038/s41416-019-0658-4

**Published:** 2019-12-10

**Authors:** Ze Zhang, Tian-En Li, Mo Chen, Da Xu, Ying Zhu, Bei-Yuan Hu, Zhi-Fei Lin, Jun-Jie Pan, Xuan Wang, Chao Wu, Yan Zheng, Lu Lu, Hu-Liang Jia, Song Gao, Qiong-Zhu Dong, Lun-Xiu Qin

**Affiliations:** 1 Department of General Surgery, Huashan Hospital, Fudan University, Shanghai, China; 2 State Key Laboratory of Oncology in South China, Collaborative Innovation Center for Cancer Medicine, Sun Yat-sen University Cancer Center, Guangzhou, China; 3 Cancer Metastasis Institute, Fudan University, Shanghai, China

**Keywords:** Cancer metabolism, Metastasis, Hepatocellular carcinoma

## Abstract

**Background:**

Mitochondrial dynamics plays an important role in tumour progression. However, how these dynamics integrate tumour metabolism in hepatocellular carcinoma (HCC) metastasis is still unclear.

**Methods:**

The mitochondrial fusion protein mitofusin-1 (MFN1) expression and its prognostic value are detected in HCC. The effects and underlying mechanisms of MFN1 on HCC metastasis and metabolic reprogramming are analysed both in vitro and in vivo.

**Results:**

Mitochondrial dynamics, represented by constant fission and fusion, are found to be associated with HCC metastasis. High metastatic HCC displays excessive mitochondrial fission. Among genes involved in mitochondrial dynamics, *MFN1* is identified as a leading downregulated candidate that is closely associated with HCC metastasis and poor prognosis. While promoting mitochondrial fusion, MFN1 inhibits cell proliferation, invasion and migration capacity both in vitro and in vivo. Mechanistically, disruption of mitochondrial dynamics by depletion of MFN1 triggers the epithelial-to-mesenchymal transition (EMT) of HCC. Moreover, MFN1 modulates HCC metastasis by metabolic shift from aerobic glycolysis to oxidative phosphorylation. Treatment with glycolytic inhibitor 2-Deoxy-d-glucose (2-DG) significantly suppresses the effects induced by depletion of MFN1.

**Conclusions:**

Our results reveal a critical involvement of mitochondrial dynamics in HCC metastasis via modulating glucose metabolic reprogramming. MFN1 may serve as a novel potential therapeutic target for HCC.

## Introduction

Hepatocellular carcinoma (HCC) is one of the most common human malignancies and the second leading cause of cancer-related mortality worldwide. Almost 50% of newly diagnosed HCC cases occur in China.^1^ Despite significant progress in HCC treatment, the prognosis of HCC patients is still far from satisfactory, mainly resulting from early metastasis and recurrence.^2^ Therefore, a new perspective of tumour metastasis mechanism in HCC is urgently needed, which may help to identify potential targets and develop effective therapies for HCC patients.

Accumulative evidence suggests that mitochondria is an important mediator of tumorigenesis and tumour progression.^3^ Mitochondria are bioenergetic, biosynthetic and signalling organelles that are critical for multiple cellular functions including growth, division, calcium homoeostasis, energy metabolism and apoptosis.^4,5^ Mitochondria exist as a dynamic network that constantly changes the morphology to meet the energy demand of cells and to adapt to the extracellular environment. Morphologically, the mitochondrial network is characterised by a mixed structure of long interconnected tubules with short isolated dot-like spheres, which is precisely regulated by two opposite processes: fusion and fission.^6^

Mitochondrial dysfunction is closely related to tumorigenesis and tumour progression, where mitochondrial dynamics plays a pivotal role.^7^ Several studies have reported dysregulated expression of mitochondrial dynamic proteins such as dynamin 1 like (DNM1L), mitofusin-1 (MFN1) and mitofusin-2 (MFN2) in lung, colon and breast cancers.^8,9^ Recent studies showed that lung cancer exhibited excessive mitochondrial fission and impaired mitochondrial fusion due to an imbalance of DNM1L/MFN expression, which is important for cell cycle progression.^10^ In addition, marked upregulation of DNM1L expression was reported to promote breast cancer metastasis via enhancing mitochondria fission.^11^ Increased mitochondrial fission was reported to promote the survival of HCC cells by facilitating autophagy and inhibiting mitochondria-dependent apoptosis.^12,13^ For HCC cells, mitochondrial fission was positively related to their metastatic capacity via modulating calcium homoeostasis.^14^

Cancer cells exhibit aberrant metabolism characterised by high glycolysis in the presence of abundant oxygen. This phenomenon, known as the Warburg effect or aerobic glycolysis, facilitates tumour growth with elevated glucose uptake and lactate production.^15,16^ The Warburg effect has now been widely accepted as a hallmark of cancer and gives rise to the development of corresponding cancer therapeutic agents. More than ten genes encoding glycolytic enzymes are found to be directly responsible for the Warburg effect.^17^

In the present study, we found notable downregulation of MFN1 expression in high metastatic human HCC cell lines and HCC patients with vascular invasions. Overexpression of MFN1 in HCC cells promotes mitochondrial fusion, and inhibits cell proliferation, invasion and migration via modulating metabolic shift from aerobic glycolysis to oxidative phosphorylation.

## Materials and methods

### Cell lines

Human embryonic kidney cell lines HEK and 293T, human HCC cell lines with genetically identical backgrounds and increasingly metastatic potential, MHCC-LM3, MHCC97-H and MHCC97-L, were obtained from the Liver Cancer Institute, Fudan University.^18^ The HCC cell lines, SMMC-7721, HepG2 and Hep3B were purchased from the Institute of Biochemistry and Cell Biology, Chinese Academy of Science (Shanghai, China). All these cell lines were cultured in Dulbecco’s modified Eagle’s medium (DMEM) (Hyclone) containing 10% foetal bovine serum (FBS) (Gibco) routinely at 37.0 °C in a humidified incubator with 5% CO_2_.

### Vectors and cell transfections

Expression vector mediated by lentivirus for human MFN1 was established. The sequence of MFN1 was amplified from cDNA library via a specific sense primer CCGGAATTCATGGCAGAACCTGTTTCTCCACT, antisense primer CGCGGATCCTTAGGATTCTTCATTGCTTGAAGGTA. Then harvested DNA was inserted into pCDH-GFP expression vector (System Biosciences).

Expression vector for MFN1 and shRNA for MFN1 were constructed as previously described. Cell transfections was also performed as previously described.^19^

### Metabolism assays

Oxygen consumption rate (OCR) and extracellular acidification rate (ECAR) were measured by using a 96-well XF or XFe extracellular flux analyzer (EFA) (Seahorse Bioscience).^20^

### Cell proliferation assays

Cell proliferation assay was carried out by using Cell Counting Kit-8 (CCK-8; Dojindo, Kumamoto, Japan). Cancer cells were seeded into 96-well plates at a density of 3 × 10^3^/100 μl cells per well (*n* = 5 for each time point) and incubated for 12, 24, 36, 48 and 72 h. After that the medium was replaced with 10% CCK-8 in complete medium. The absorbance at 450 nm was measured after incubation for 2 h. Each experiment was repeated three times on each condition.

### Colony formation assays

Cancer cells were seeded into six-well plates at a density of 1 × 10^3^ cells in each well and incubated for 2 weeks. After that, cells were fixed in 4% formalin for 20 min and stained with 1% crystal violet for 20 min. The number of colonies was counted after the plates were being stained. Each experiment was repeated three times.

### Wound-healing assays

Monolayers of cells were plated in 12-well plates and then scratched with the tip of a 20-μL pipette and washed several times with PBS (Hyclone) until dislodged cells were removed clearly. Tumour cells were cultured in DMEM medium free from FBS. The healing status of wound area was photographed at 0 h and 24th/48th/72nd hour after scratching.

### Transwell assays

Transwell assays with and without Matrigel were carried out by using 24-well plate with Transwell chambers with 8-µm pores (BD Pharmingen). The bottom chamber was filled with DMEM medium with 20% FBS as a chemoattractant. Fifty thousand cells for migration assay and 10 × 10^4^ cells for invasion assay were seeded into the upper chamber maintained in medium free from serum. Cells migrated to the underside of the membrane were fixed with 4% formaldehyde, stained with 1% crystal violet and counted with light microscope (×100, Leica).

### Animal studies

All experimental procedures involving animals were approved by The Animal Care and Use Committee of Huashan Hospital of Fudan University (Shanghai, China). All mice (BALB/c nu/nu, 18.69 ± 0.69 g), under the age of 5 weeks (±3 days), were obtained from Shanghai Slac Laboratory Animal Co. and fed in a specific pathogen-free (SPF) vivarium under standard conditions. Briefly, mice were housed in a 12-h light/dark cycle, temperature- (22 ± 1 °C) and humidity- (55 ± 5%) controlled room and were allowed free access to water and a maintenance diet. All cages contained sterilised wood shavings. Prior to any surgical procedure, all mice were given intraperitoneal (i.p.) injection of Pentobarbital Sodium (40 mg/kg; Sigma Aldrich, St. Louis, MO, USA) as anaesthesia because of its effectiveness and safety. All experimental procedures were conducted in the SPF laboratory during light phases. Mice were finally killed by cervical dislocation.

To construct subcutaneous xenograft tumour model, four HCC cell lines including EV MHCC97-H, OE-MFN1 MHCC97-H, sh-Ctrl HepG2 and sh-MFN1 HepG2 (1 × 10^6^ cells suspended in 100 μl of Matrigel and PBS) were injected into the right flanks of mice that were randomly allocated into four groups (*n* = 5 mice/group) after anaesthesia. Tumour volume was monitored weekly with a calliper until mice were killed after 6 weeks. Tumour volume was calculated by the formula: a × b^2^/2 (where a and b separately represent the largest and smallest tumour diameters).^21^ All the tumours were collected. RNA was extracted from fresh tumour tissues and the rest of the tumours were fixed in 4% formalin, and then embedded in paraffin. Consecutive sections of tumour tissues were applied by immunohistochemistry staining.

For orthotopic model, tumours from subcutaneous xenograft tumour model of four groups were minced into 1–2 mm^3^ sections and inoculated into the left liver lobes of mice that were randomly allocated into four groups (*n* = 5 mice/group) after anaesthesia. Mice were killed after 7 weeks.^21^ Tumours, livers and lungs of all mice were collected and fixed in 4% formalin, and then embedded in paraffin. Consecutive sections of lung tissue were stained with haematoxylin and eosin. The number of lung metastasis was calculated and evaluated independently by two pathologists.

### Clinical samples, tissue microarray (TMA) and immunohistochemistry (IHC)

We applied IHC staining analysis on a cohort consisting of 87 HCC patients, by dividing them into two groups, high MFN1 expression (H-MFN1) and low MFN1 expression (L-MFN1). Clinical samples from patients were obtained after acquiring their consent in accordance with the protocol approved by the Ethics Boards of Huashan Hospital of Fudan University (Shanghai, China).

Formalin-fixed and paraffin-embedded tissues were used to construct TMA as previously described.^21^ Four-micron core biopsies from the donor blocks were taken and transferred to the recipient paraffin block at predefined array positions and constructed 87 cases of TMA blocks in this study. IHC staining was performed as described previously.^18^ After deparaffinisation, rehydrating and antigen retrieval, primary antibodies were added to slides, incubated at 4 °C overnight, and followed with incubation with secondary antibody (Dako Cytomation, Glostrup, Denmark) at 37 °C for 30 min. Staining was performed with DAB and counterstaining was operated with haematoxylin.

### Evaluation of IHC scores

Scoring for MFN1 staining was conducted as previous described,^22^ briefly using percentage score × staining intensity score. The percentage of positive-staining cells: 0–5% scored 0, 6–25% scored 1, 26–50% scored 2, 51–75% scored 3 and more than 75% scored 4; staining intensity: no staining scored 0, weakly staining scored 1, moderately staining scored 2 and strongly staining scored 3.

### Immunofluorescence (IF)

After incubating with 100 nM MitoTracker Deep Red in DMEM medium with 10% serum, 5 × 10^3^ HCC cells were plated into 24-well plate containing slide glasses of 12 mm in diameter and then fixed in 4% paraformaldehyde for 20 min. After washing with PBS three times, cells were incubated in 1% BSA at room temperature for 1 h. After washing with PBS three times, samples were counterstained with DAPI 100 μg/ml in PBS for 10 min. Finally, the slides were sealed on a slide mounted in antifade reagent (Bevotime, China). Fluorescence were detected by using confocal fluorescent microscope (Nikon, Tokyo, Japan).

### Mitochondrial length

Mitochondrial length was assessed by staining with MitoTracker Deep Red and performed as described before. Mitochondrial length was measured by tracing the mitochondria using ImageJ software. Mitochondrial length was either binned into different categories (<1 mm, fragmented; 1–2 mm, intermediated; >2 mm, elongated) or taken as an average.

### Western blotting

Western blotting was carried out as described before.^22^ Total protein was extracted by RIPA buffer containing protease cocktail inhibitor. All protein samples were quantified. Protein samples were separated by sodium dodecyl sulfate polyacrylamide gel electrophoresis (SDS-PAGE) and then transferred onto polyvinylidene fluoride (PVDF) membranes. After blocking with 5% skim milk in TBS-T (BD Pharmingen), PVDF membranes were incubated with the primary antibody overnight at 4 °C and then with the secondary antibody for 1 h at room temperature. The following antibodies were used: anti-MFN1 (1:1000, Abcam), anti-MFN2 (1:1000, Abcam), anti-Opa1 (1:1000, Abcam), anti-DNM1L (1:1000, Abcam), anti-MFF (1:1000, Abcam), anti-TOM20 (1:1000, Abcam), anti-E-cadherin (1:1000, Abcam), anti-N-cadherin (1:1000, Abcam), anti-Snail (1:1000, Abcam), anti-Twist (1:1000, Abclonal), anti-Zeb1 (1:1000, Abclonal), anti-Slug (1:1000, Abclonal) anti-Vimentin (1:1000, Abcam) and anti-β-actin (1:1000, Abcam). Protein bands were detected by using image acquisition using ImageQuant™ LAS 4000 (GE Healthcare Life Sciences).

### RNA isolation, reverse transcription and quantitative real-time polymerase chain reaction (qRT-PCR)

RNA of cell lines and frozen tissue was isolated using Trizol reagent (Invitrogen). After quantification, complementary DNA synthesis was performed using PrimeScript reverse transcriptase reagent kit (Takara) according to the manufacturer’s directions.

Quantified real-time PCR was performed using SYBR Green PCR Master Mix (Takara) and then detected by ABI PRISM 7900 Sequence Detection System (Applied Biosystems). The results were normalised to β-actin for mRNA measurement. All the primers are listed in Supplementary Table 1. Each experiment was repeated six times on each condition.

### Statistical analysis

Statistical analyses were carried out using Statistical Package for Social Sciences Version 21.0 (SPSS 21.0) and Graphpad Prism^®^ 7.0. The analysis of variance (ANOVA) test was used to compare mean values among three or more groups, whereas independent-sample two-sided Student’s t test was used to compare two groups with normal distribution data. The correlation analysis was determined by Pearson. Kaplan–Meier survival analyses were used to estimate the overall survival and disease-free survival, and the log-rank test was used to assess the differences. All statistics were two sided and *p* < 0.05 was considered statistically significant.

## Results

MFN1 is identified as a leading downregulated gene in mitochondria dynamics closely associated with HCC metastasis.

**Fig. 1:**
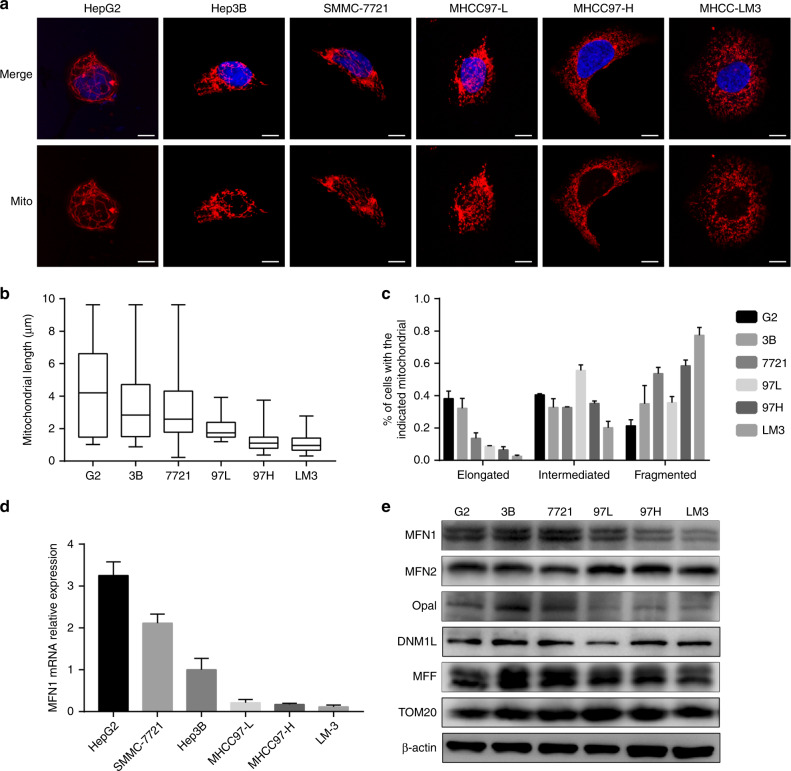
Mitochondrial dynamics in HCC cell lines with different metastatic potential. Mitochondria fusion is less frequent in HCC cells of higher metastatic capacity and correlated to MFN1. **a** Representative immunofluorescence images under a confocal microscope of mitochondrial morphology in HepG2 (G2), Hep3B (3B), SMMC-7721 (7721), MHCC97-L (97L), MHCC97-H (97H) and HCC-LM3 (LM3) cell lines stained with MitoTracker Deep Red. Scale bars: 10 µm. High metastatic HCC cells like MHCC97-H and LM3 intended to have more fragmented and less fused mitochondria, while low metastatic HCC cells like HepG2 exhibited an opposite morphology. **b**, **c** Quantified analysis on mitochondrial length in different HCC cells and ratio analysis based on quantified mitochondria length confirmed the morphologic alterations in IF images. *n* = 3, mean ± SEM. **d**, **e** qRT-PCR (*n* = 6, mean ± SEM) and western blot analysis for mRNA and protein levels of mitochondrial dynamic mediators. Mitochondria fusion mediator MFN1 showed most significant changes in protein level among HCC cells with different metastatic potential, consistent with mRNA levels.

To evaluate mitochondrial dynamics in human HCC cell lines with different metastatic potential, we applied IF technique to manifest mitochondrial morphology. The MitoTracker Deep Red showed an explicit phenomenon that HCC cell lines with high metastatic potential, like MHCC97-H and HCC-LM3, tended to have more fragmented mitochondria, while those with low metastatic potential, like HepG2 and Hep3B, shared much more elongated mitochondria (Fig. 1a). Similar results were also observed by fluorescence in human HCC cell lines with different metastatic potential (Fig. 1b, c). To further identify the key regulators of mitochondrial dynamics, we performed qRT-PCR (Fig. 1d) and western blot (Fig. 1e, Fig. S1a–d) for such candidates including MFN1, MFN2, Opa1 and MFF. MFN1 showed significant variations among HCC cell lines with different metastatic potential, in both mRNA and protein levels. High metastatic HCC cell lines like MHCC97-H and HCC-LM3, had significantly decreased expressions of MFN1, which is consistent with their more fragmented mitochondria (Fig. 1a–e). These results suggested that mitochondria in high metastatic HCC cell lines prefer a dynamics imbalance towards fission, which is accompanied by MFN1 downregulation.

### Downregulation of MFN1 is associated with vascular invasion and poor outcome of HCC patients

**Fig. 2:**
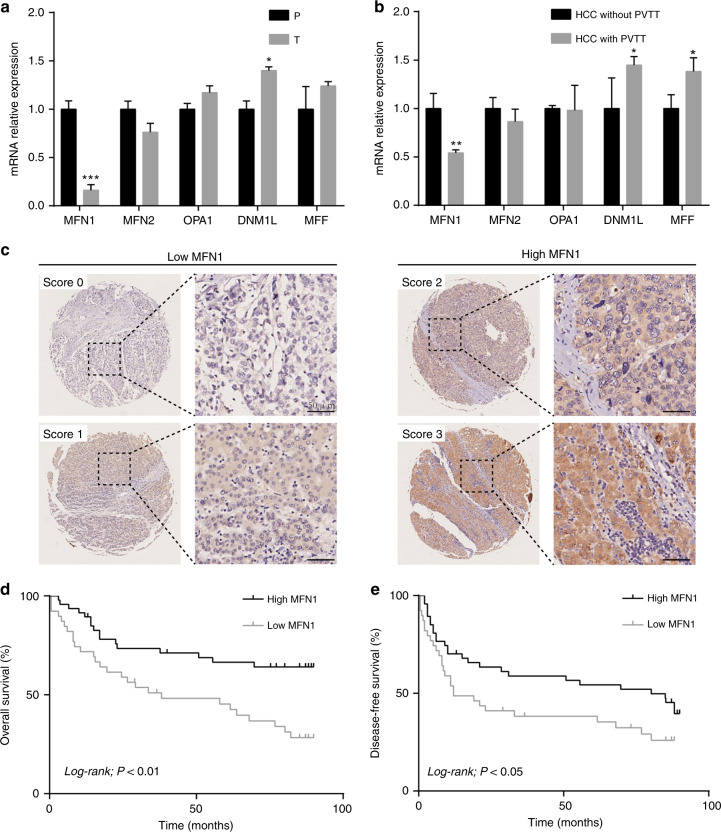
Low MFN1 level in human HCC was correlated with vascular invasion and poor prognosis. **a** qRT-PCR analysis for MFN1 expression levels in 34 HCC samples between tumour and peritumour mesenchyme tissues indicated that MFN1 expression was greatly inhibited in tumour tissues compared with para-tumour tissues. *n* = 6, mean ± SEM, ***P < 0.001, **P < 0.01, *P < 0.05. **b** qRT-PCR analysis for MFN1 expression levels in 20 HCC patients with and without portal vein tumour thrombosis (PVTT) indicated that for patients with PVTT MFN1 expression decreased. *n* = 6, mean ± SEM, ***P < 0.001, **P < 0.01, *P < 0.05. **c** Representative immunohistochemical (IHC) staining images of MFN1 in HCC tissue microarrays. Scale bar: 100 µm. **d**, **e** Kaplan–Meier analysis of overall survival (OS) and disease-free survival (DFS) in HCC patients classified by expression levels of MFN1 showed that MFN1 low expression contributes to poor prognosis.

To further understand the regulatory role of mitochondrial dynamics in HCC, we compared expression levels of mitochondrial dynamics-related genes between tumour and peritumour mesenchyme tissues by qRT-PCR in 34 HCC samples (Fig. 2a). MFN1 expression was greatly decreased in tumour tissues compared with para-tumour tissues. Correlation analysis between patients’ clinical information and MFN1 expression levels elaborated that MFN1 expression was negatively related to TNM stage and portal vein tumour thrombosis (PVTT) frequency (Table S1, Fig. S2a, b). PVTT from HCC was apparently linked to high recurrence and intrahepatic metastasis. To define the specific changes in candidate mitochondrial dynamics regulators associated with metastatic progression, we checked their expression using ten pairs of HCC samples by qRT-PCR. The expression levels of three genes, including MFN1, DNM1L and MFF, were significantly different between HCC with and without PVTT, among which the difference of MFN1 was the greatest (Fig. 2b). In these patients with PVTT, we observed that HCC cells with low staining degrees particularly tend to invade into vascular (Fig. S2b). In addition, Kaplan–Meier analysis showed that patients with low MFN1 expression had obviously a poorer prognosis in both disease-free survival (DFS) and overall survival (OS) than those with high MFN1 expression (log rank *P* < 0.05 and < 0.01, respectively, Fig. 2c–e).

### MFN1 inhibits HCC proliferation and metastasis via mitochondria fusion in vitro and in vivo

**Fig. 3:**
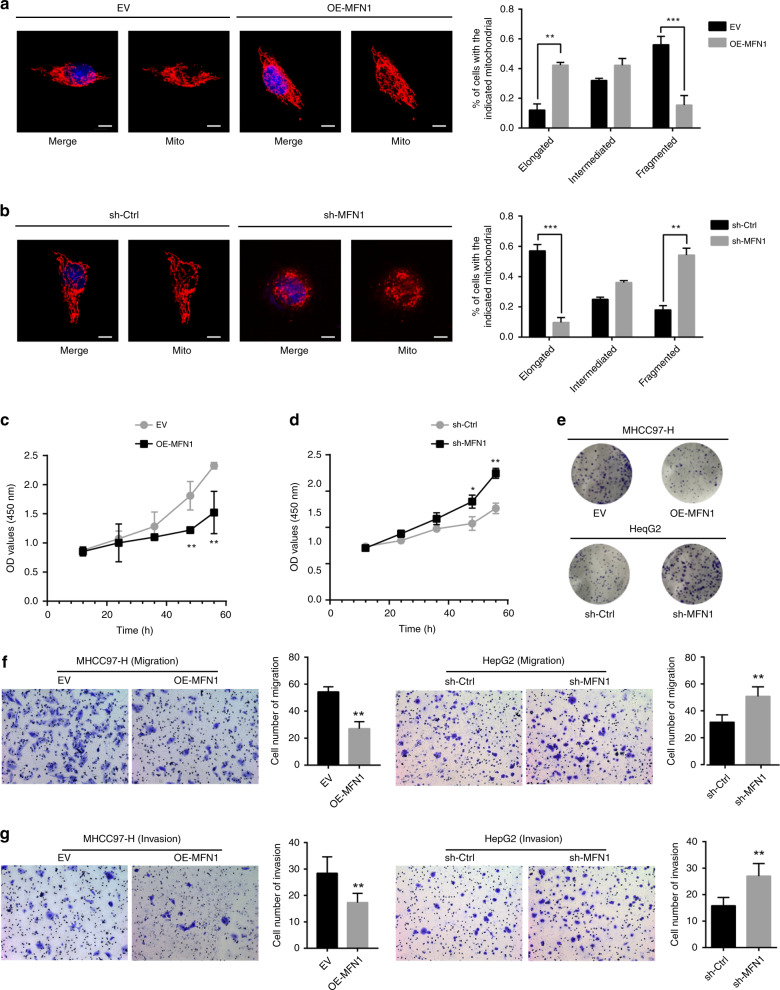
Increased mitochondrial fusion mediated by MFN1 inhibits migration and invasion of HCC cells in vitro. **a**, **b** Representative immunofluorescence images under a confocal microscope of mitochondrial morphology of OE-MFN1 97H cells and HepG2 cells. Scale bars: 10 μm. MFN1 overexpression leads to increased mitochondria fusion, while knockdown of MFN1 results in mitochondria fragmentation. Quantified analysis on mitochondrial length and ratio analysis based on quantified mitochondria length OE-MFN1 97H cells and sh-MFN1 HepG2 cells confirmed the morphologic changes in IF. *n* = 3, mean ± SEM, ***P < 0.001, **P < 0.01, *P < 0.05. **c**, **d** Cell proliferation assay was assessed by CCK-8 assay in OE-MFN1 97 H cells and sh-MFN1 HepG2 cells. Overexpression of MFN1 inhibited 97H cell proliferation, while knockdown of MFN1 promoted HepG2 cells proliferation. OD, absorbance degrees. *n* = 3, mean ± SEM, ***P < 0.001, **P < 0.01, *P < 0.05. **e** Images on plate clone formation assay in OE-MFN1 97H cells and sh-MFN1 HepG2 cells. The results were consistent with cell proliferation assay. **f**, **g** Images and quantified column figures on Transwell assay in OE-MFN1 97H cells and sh-MFN1 HepG2 cells. Overexpression of MFN1 inhibited migration and invasion of 97H cells, while knockdown of MFN1 promoted migration and invasion of HepG2 cells. *n* = 3, mean ± SEM, ***P < 0.001, **P < 0.01, *P < 0.05.

To explore the functions of mitochondrial fusion and MFN1 in HCC cells, we performed both in vitro and in vivo gain- and loss-of-function studies. We established MHCC97-H cell line with stable overexpression of MFN1 (OE-MFN1) and MFN1-knockdown HepG2 cell line (sh-MFN1) (Fig. S3a–d). Overexpression of MFN1 led to increased fusion of mitochondria (elongated mitochondria) (Fig. 3a). On the contrary, knockdown of MFN1 resulted in more mitochondrial fragmentation (Fig. 3b). Furthermore, overexpression of MFN1 significantly inhibited proliferation (Fig. 3c), clone formation (Fig. 3e), migration and invasion (Fig. 3f, g, Fig. S3e) of MHCC97-H cells in vitro, while knockdown of MFN1 exhibited an opposite effect in HepG2 (Fig. 3d–g, Fig. S3f). As epithelial-to-mesenchymal transition (EMT) is suggested to be closely associated with acquisition of pro-invasive capacities in tumour,^23^ we wondered whether MFN1 plays important roles in EMT of HCC cells. As expected, MFN1 induced an increased expression of the epithelial marker E-cadherin, and reduced expression of mesenchymal markers including N-cadherin, vimentin as well as snail (Fig. S4a, left panel). Consistently, knockdown of MFN1 in HepG2 led to the opposite effect (Fig. S4a, right panel).

**Fig. 4:**
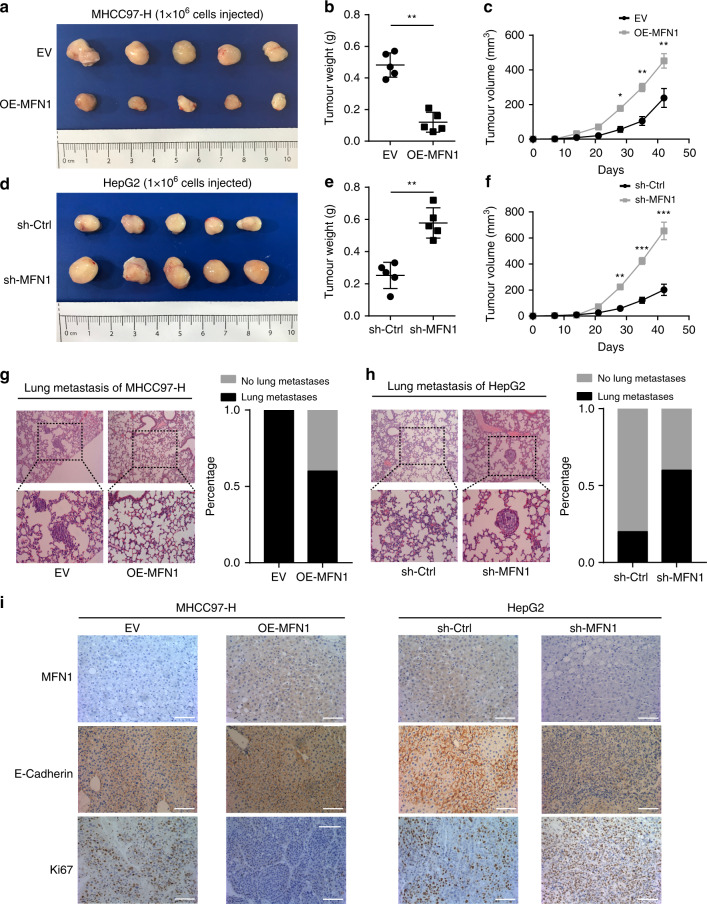
Increased mitochondrial fusion mediated by MFN1 inhibits proliferation and metastasis of HCC cells in vivo. **a–f** The effects of MFN1 gain- or loss of function on the dynamic change of tumour volume in subcutaneous xenograft models (**a**, **d**). Quantitative analysis (**b**, **c**, **e**, **f**) demonstrated that overexpression of MFN1 inhibited subcutaneous tumour growth, while knockdown of MFN1 promoted subcutaneous tumour growth. *n* = 5, mean ± SEM, ***P < 0.001, **P < 0.01, *P < 0.05. **g**, **h** The effects of MFN1 gain- or loss of function on the dynamic change of the spontaneous lung metastasis in orthotopic xenograft models. Representative H&E staining images of lung tissues (left) and the percentage of nude mice with lung metastasis (right) from five mice per group were shown. MFN1 overexpression inhibited lung metastasis of 97H cells, while knockdown of MFN1 promoted lung metastasis of HepG2 cells. *n* = 5, mean ± SEM, ***P < 0.001, **P < 0.01, *P < 0.05. **i** IHC staining image of MFN1, E-cad and Ki67 in xenografted tumour from nude mice. MFN1 staining validated the efficiencies of MFN1 overexpression in 97 H cells and MFN1 knockdown in HepG2 cells. E-cadherin staining confirmed positive correlation between E-cadherin and MFN1. Ki67 staining verified that MFN1 negatively modulates proliferation of HCC cells. Scale bar: 100 μm.

To verify the role of MFN1 in vivo, we constructed subcutaneous and orthotopic xenograft models. In subcutaneous xenograft models, the average volume of MHCC97-H tumour in the MFN1 transfection group was significantly smaller than the control group (Fig. 4a–c). On the other hand, downregulation of MFN1 could significantly promote in vivo tumour growth with much larger tumour sizes than control in xenograft models of HepG2 cells (Fig. 4d–f). In orthotopic xenograft models, MHCC97-H or HepG2 subcutaneous xenografts were isolated and implanted into the liver to establish orthotopic xenografts. The percentage of nude mice with lung metastasis and the total numbers of lung metastatic lesions in the MFN1 overexpression group were much lower than those in the control group (Fig. 4g, Fig. S4d). However, an increase in the percentage of lung metastasis was observed in nude mice bearing orthotopic xenografts from sh-MFN1-HepG2 cells (Fig. 4h), though the total numbers of lung metastatic lesions in MFN1-knockdown group were not significantly increased (Fig. S4e).

We further detected the expression of MFN1, E-cadherin and Ki67 in xenograft tumours. Consistent with the change of EMT-related protein expression in OE-MFN1 MHCC97-H and sh-MFN1 HepG2, we observed obviously positive correlation between E-cadherin and MFN1 in xenograft tumours from OE-MFN1 MHCC97-H cells and sh-MFN1 HepG2 cells. The expression level of Ki67 was low in OE-MFN1 MHCC97-H xenograft tumours and high in sh-MFN1 HepG2 xenograft tumours (Fig. 4i). These in vitro and in vivo gain- and loss-of-function studies indicate that MFN1 inhibits HCC progression via controlling mitochondria fusion.

### MFN1 modulated glucose metabolic reprogramming in HCC

**Fig. 5:**
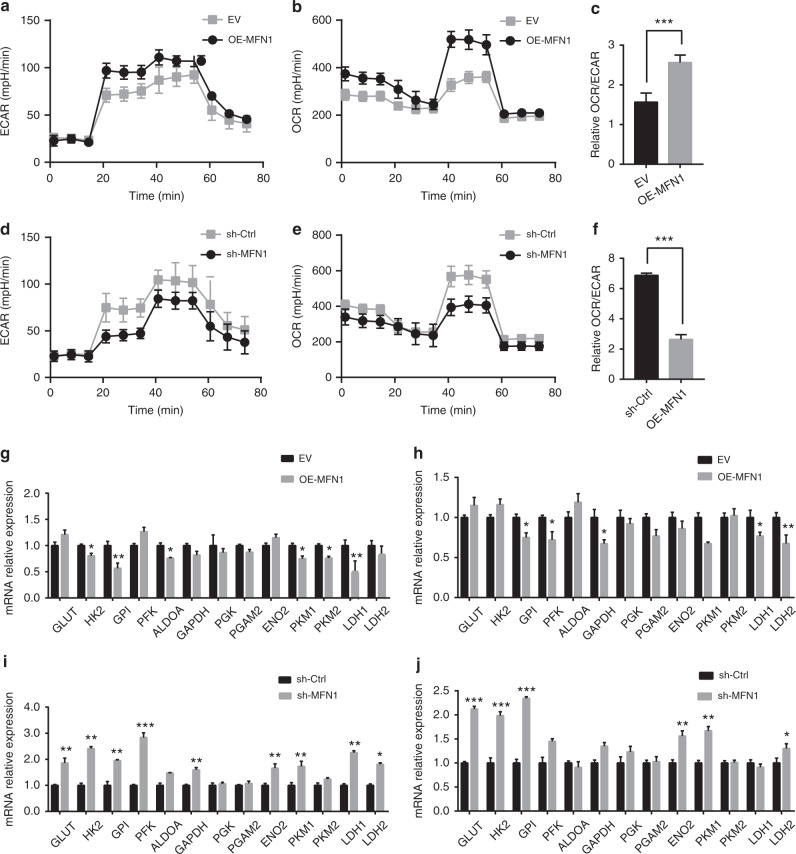
Metabolic reprogramming in HCC cells mediated by MFN1. **a–f** Sea-horse metabolism analysis on overexpression and knockdown of MFN1 HCC cells. The ratio of OCR/ECAR increased significantly in OE-MFN1 97H cells, stating increased O_2_ consumption compared with less lactic acid production, while the ratio of OCR/ECAR decreased significantly in knockdown HepG2 cells. *n* = 3, mean ± SEM, ***P < 0.001. **g–j** Aerobic glycolysis-related enzymes were detected using qRT-PCR in OE-MFN1 97H cells, sh-MFN1 HepG2 cells and the xenograft tumours. Most enzymes involved in aerobic glycolysis were downregulated in OE-MFN1 97 H cells (**g**) and their xenograft tumour (**h**) and upregulated in sh-MFN1 G2 cells (**i**) and the xenografts (**j**). *n* = 6, mean ± SEM, ***P < 0.001, **P < 0.01, *P < 0.05.

Since mitochondria play an essential role in cell metabolism and energy production, we hypothesised that disrupted mitochondria dynamics might influence metabolic reprogramming in HCC cells. We used Seahorse platform to monitor the metabolism state of OE-MFN1 MHCC97-H cells and sh-MFN1 HepG2 cells (Fig. 5a–f). We found that MFN1 could induce a glucose metabolic shift from aerobic glycolysis to oxidative phosphorylation (Fig. 5a–c), while aerobic glycolysis was promoted when MFN1 was knocked down in HepG2 cells (Fig. 5d–f). To better understand this metabolic shift induced by MFN1 in HCC, we measured the expression of metabolic enzymes in glucose metabolism by qRT-PCR. The representative enzymes crucial for oxidative phosphorylation were found highly expressed in OE-MFN1 MHCC97-H cells, while the expression levels of key metabolic enzymes involved in aerobic glycolysis significantly increased in sh-MFN1 HepG2 cells (Fig. 5g–j, Fig. S5a–d). These results suggest that MFN1 modulates the glucose metabolic reprogramming in HCC cells.

### A decrease in anabolic glycolysis is required for the anti-metastatic effect of MFN1

**Fig. 6:**
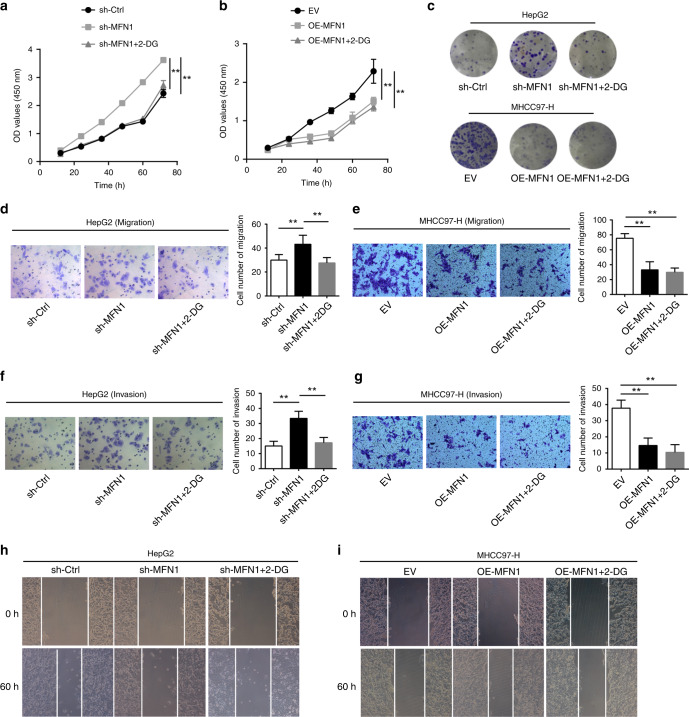
MFN1 influences HCC cells proliferation, invasion and migration via metabolic reprogramming. **a–c** Proliferation assay (**a**) and plate clone formation assay (**c**) showed that 2-DG inhibited the proliferation of sh-MFN1 HepG2 cells. But 2-DG did not affect proliferation capacity in OE-MFN1 97H cells (**b**). *n* = 3, mean ± SEM, ***P < 0.001, **P < 0.01, *P < 0.05. **d–i** Transwell assay (**d**, **e**, **f**, **g**) and wound-healing assays (**h**, **i**) confirmed that 2-DG could reverse the effect of sh-MFN1 to retrain migration and invasion of HepG2 cells. *n* = 3, mean ± SEM, ***P < 0.001, **P < 0.01, *P < 0.05.

In order to ascertain whether glucose metabolic shift induced by depletion of MFN1 directly affects the function of HCC cells, we used 2-Deoxy-d-glucose (2-DG) to selectively block aerobic glycolysis in HCC cells. 2-DG is a glycolysis inhibitor and competitively inhibits the production of glucose-6-phosphate. HepG2 cells treated with aerobic glycolysis inhibitor 2-DG no longer exhibited high proliferation (Fig. 6a), clone formation (Fig. 6c), migration (Fig. 6d, h) and invasion (Fig. 6f) induced by depletion of MFN1. Furthermore, 2-DG could inhibit EMT enhanced by MFN1 knockdown in HCC cells (Fig. S6a). Instead, treatment of 2-DG showed no effects on the function of HCC cells with overexpression of MFN1 (Fig. 6b, c, e, g, i). Together, we concluded that metabolic shift against aerobic glycolysis would restrain HCC proliferation, invasion and migration. This shift was achieved by MFN1-mediated alteration of mitochondria dynamics.

## Discussion

As a milestone in cancer metabolism research, aerobic glycolysis is first described by Otto Warburg. Warburg effect had long been interpreted as an adaptation for tumour under stringent conditions with impaired mitochondrial respiration.^15^ However, growing evidence begins to reshape the view on cancer metabolism reprogramming. It is known that mitochondria are actively functioning in some types of cancer and contribute to cancer progression.^24–26^ Currently, metabolic reprogramming has been regarded as a general hallmark of malignant tumour.

Mitochondria play a central role in coordinating the physiology and metabolism of most eukaryotic cells. Mitochondria are morphologically heterogeneous organelles and undergo constant fission and fusion that is closely related to differed functional states.^27,28^ Mitochondrial dynamics has been recognised as an important biological process in carcinogenesis and cancer progression.^29–31^ Mitochondrial dynamics was reported to regulate the migration and invasion of breast cancer.^11^ Also, mitochondrial fission promotes autophagy, preventing cancer from apoptosis.^12^ In this study, we demonstrated that mitochondrial dynamics was associated with HCC metastasis. High metastatic HCC displayed excessive mitochondrial fission with more fragmented mitochondria.

It is widely known that mitochondrial fission and fusion balance is regulated by several key regulators, including MFN2, DNM1L, OPA1 and MFF.^32–34^ In our current research, we found that these regulators of mitochondrial dynamics showed differed metastatic potential in HCC, and that MFN1 is a leading downregulated gene in mitochondria dynamics closely associated with HCC metastasis. Furthermore, MFN1 was identified as a tumour-suppressor gene, promoting the mitochondrial fusion to suppress the invasion and metastasis both in vitro and in a xenograft model in vivo. The clinical data support the in vitro and xenograft findings showing that downregulation of MFN1 is associated with vascular invasion and poor prognosis of HCC patients. The data would be more intact and convincing to reveal the underlying connection between MFN1 expression and HCC metastasis if we could compare expression of MFN1 in primary and lung metastatic tissues from patients. According to the current clinical practice guidelines, surgical treatment is not recommended to HCC patients with lung metastasis. Thus, it is not feasible for us to acquire samples from these patients. We hope to collect such samples for deeper-insight research in our future study. Previous studies demonstrated that increased DNM1L expression upregulated mitochondrial fission to promote breast cancer progression.^35^ In our study, DNM1L was also slightly upregulated in HCC patients with PVTT but not in HCC cell lines with high metastatic potential. Beyond the above, the function of DNM1L on regulating mitochondrial fission relies on phosphorylation of two sites (serine 616 and serine 637);^36^ it requires further solid evidence to elucidate the actual function that DNM1L carries in HCC.

Cancer metastasis is a multistep process, influenced by different factors. EMT usually represents early steps of metastasis such as circulating tumour cells (CTC) formation, which is often linked to a poor prognosis in many types of malignancies including cholangiocarcinoma, gastric cancer, breast cancer and so on.^37–40^ Previous studies have demonstrated that EMT is vital as a metastatic driver in different cancers including HCC. In the present study, we found that HCC cells with MFN1 depletion showed E-cadherin downregulation and increased mesenchymal markers, all of which are critical regulators of EMT. These results were further confirmed by in vivo evidences that MFN1 strongly decreased the metastatic potential of HCC cells along with changes in their expression pattern of E-cadherin. Furthermore, we found the correlation between mitochondria dynamics-mediated metabolic reprogramming and EMT activation. We hope that this regulatory mechanism can be elaborated in the future.

Cancer cells exhibit a featured glucose metabolic shift to increased aerobic glycolysis compared with normal tissues. Evidence has been accumulated that key metabolic enzymes involved in aerobic glycolysis and their modifications regulate cancer metastasis.^41,42^ Furthermore, metabolic reprogramming resulted from other processes also influences the metastasis.^43^ As mitochondria take the central role in energy metabolism including glucose metabolism, metabolic shift must be closely connected with mitochondrial function, which is largely dependent on mitochondrial dynamics. In the present study, we demonstrated that MFN1 modulated HCC metastasis by metabolic shift from aerobic glycolysis to oxidative phosphorylation, which was attributed to mitochondrial fusion. Treatment with aerobic glycolysis inhibitor 2-DG could significantly suppress the effects induced by MFN1 depletion. We also confirmed that the expressions of glycolysis-related genes were influenced by mitochondrial dynamics. Though we did not clarify the specific mechanism on how mitochondrial dynamics influenced expression of glycolysis-related genes, according to multiple researches published previously, MFN1/2 can modulate Ca^2+^ buffering through ER–mitochondria tethering activity, thereby regulating nuclear translocation and transcriptional activity of multiple genes.^4,44,45^ This may possibly explain potential modulation mechanism on glycolysis-related genes induced by MFN1. Overall, these findings may provide innovative ideas for integrating how metabolic reprogram influences the metastatic process.

In conclusion, we found that MFN1 suppressed HCC malignancy via driving the balance of mitochondrial dynamics from fission to fusion, which mediated metabolic shift from aerobic glycolysis to oxidative phosphorylation. MFN1 may serve as a novel potential target for new metabolic therapies in HCC.

## Supplementary information

Supplementary

## Data Availability

All data and materials generated during and/or analysed during this study are available from the corresponding author on reasonable request.
