# Supplementary material for: MFN1-dependent alteration of mitochondrial dynamics drives hepatocellular carcinoma metastasis by glucose metabolic reprogramming
Source: Br J Cancer. 2019 Dec 10;122(2):209–20. doi: 10.1038/s41416-019-0658-4 (PMC7052272; doi:10.1038/s41416-019-0658-4)
Supplement: Supplementary file 1 — Supplementary [file 41416_2019_658_MOESM1_ESM.docx]

/Para><Para ID="Par2" OutputMedium="Online"><Figure ID="Figa" Float="Yes" Category="Standard"><MediaObject><ImageObject Color="BlackWhite" Format="PNG" Rendition="HTML" Type="Linedraw" FileRef="42003_2024_6834_Figa_HTML.png" Height="10" Resolution="120" Width="20"/></MediaObject></Figure></Para></Abstract><Abstract ID="Abs2" Language="En" Type="Short---------graphical abstractFig. S1 Mitochondria dynamic related genes mRNA level in HCC cell lines with different metastatic potential. **(a-d)** Message RNA levels of other mitochondria dynamic related genes including DNM1L **(a)**, Opa1 **(b)**, MFN2 **(c)** and MFF **(d)** were detected in different HCC cell lines. N=6, Mean ± SEM.


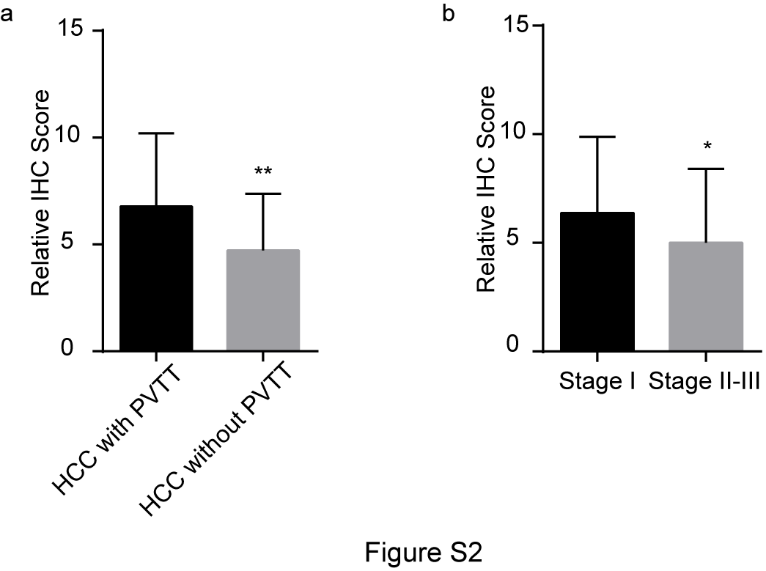


Fig. S2 MFN1 IHC scores in HCC samples. **(a, b)** Chi-square test analysis on 48 HCC patients in forms of column figure stated that MFN1 expression was negatively corrected with TNM stage and PVTT occurrence. Mean ± SEM. **, P<0.01; *, P<0.05.


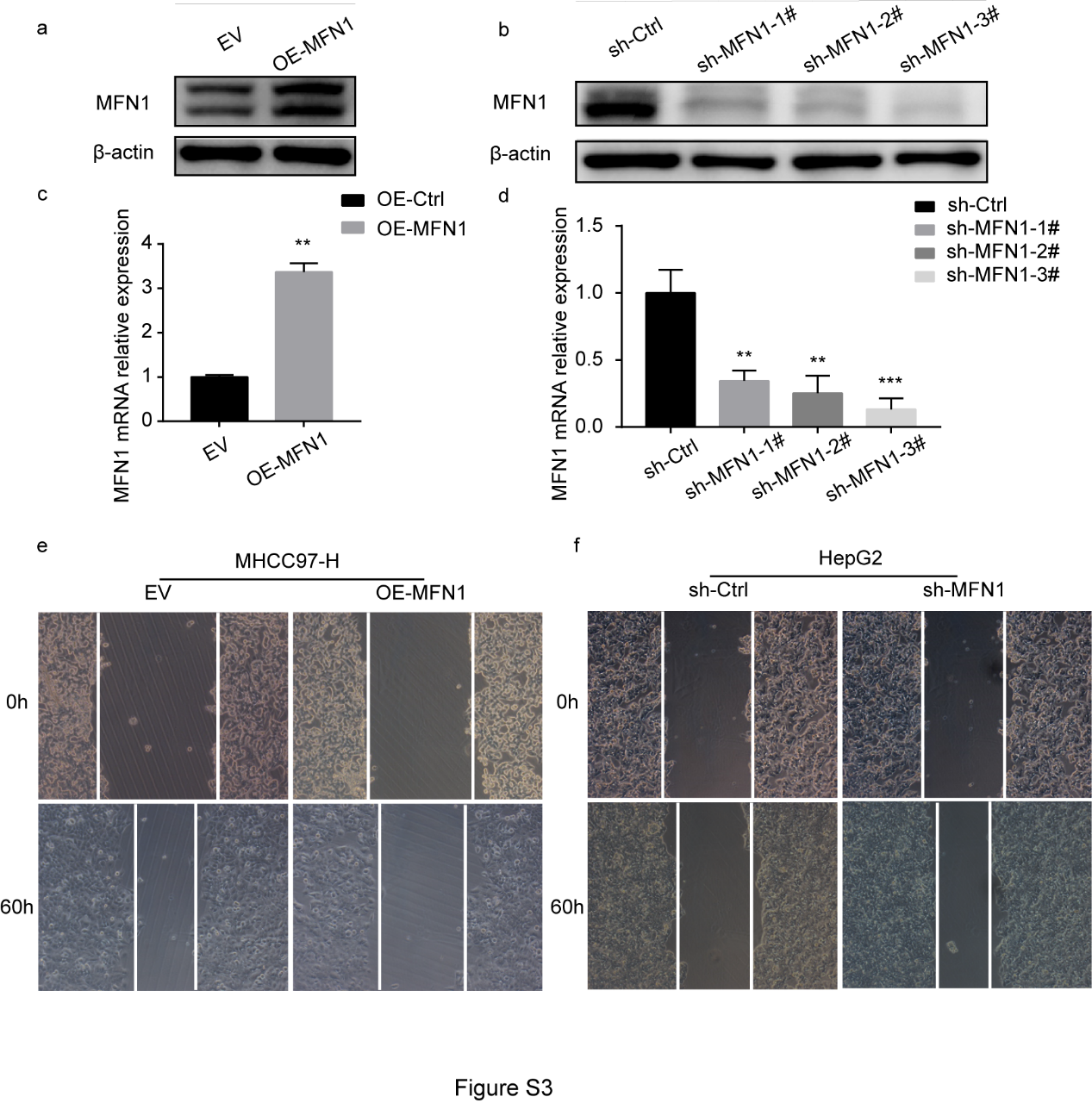


Fig. S3 Efficiencies of MFN1 overexpression in MHCC97-H cells and MFN1 knockdown in HepG2 cells. **(a, b)** Western blot analysis confirmed the efficiencies of overexpression in 97 cells and knockdown MFN1 in HepG2 cells in protein levels. EV, empty vector; OE, overexpression; Sh, small hairpin RNA. **(c, d)** QRT-PCR analysis confirmed the efficiencies of overexpression in 97 cells and knockdown MFN1 in HepG2 cells in mRNA levels. N=6, Mean ± SEM, ***, P<0.001; **, P<0.01; *, P<0.05. **(e, f)** Wound healing assay in MFN1 overexpressed MHCC97H cells and MFN1 knockdown G2 cells. Overexpression MFN1 inhibited 97H cell migration while knockdown MFN1 promoted G2 cells migration.

**
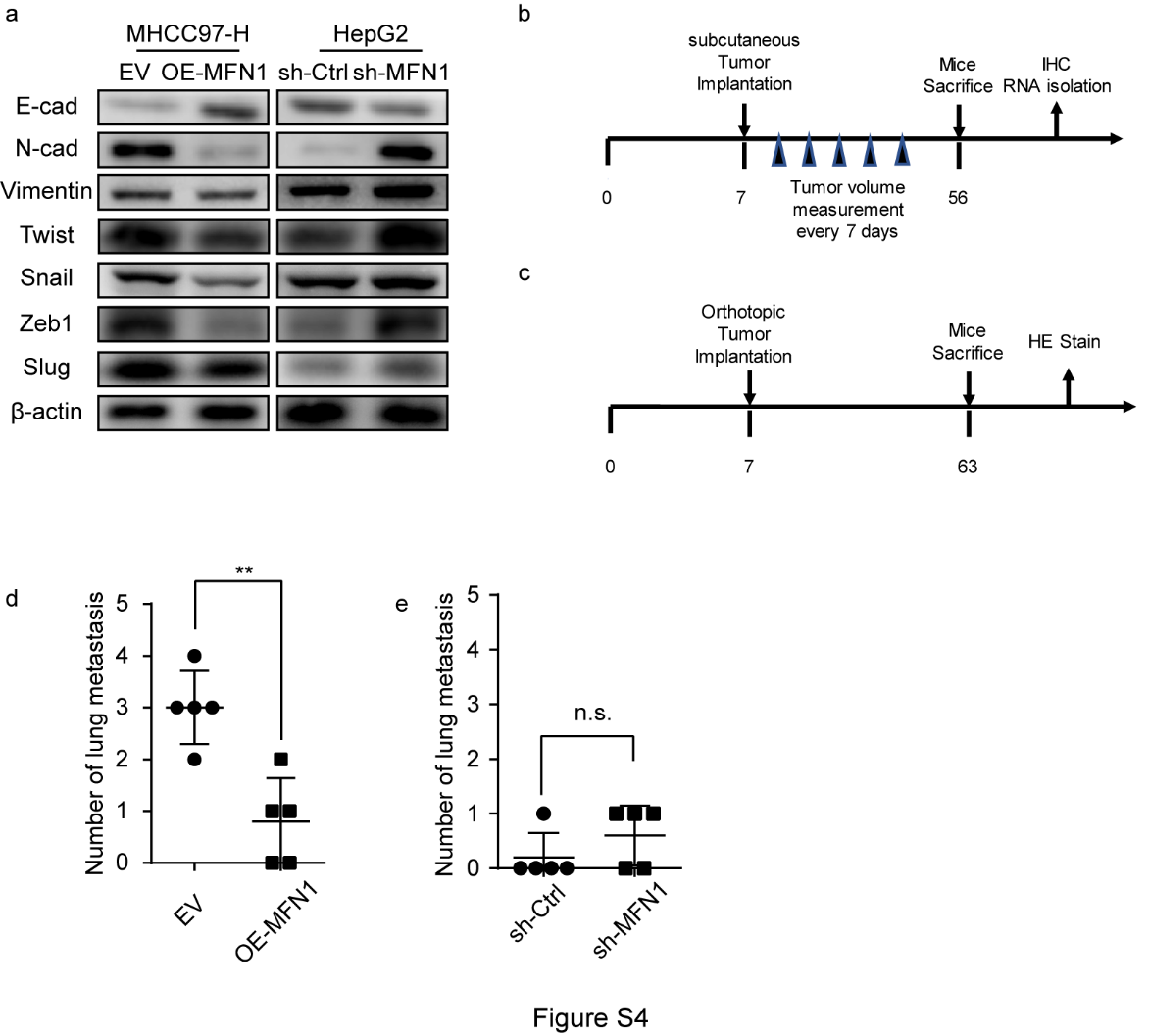
**

Fig. S4 **(a)** Western blot analysis for protein levels of EMT related marker proteins including E-cadherin, N-cadherin, Vimentin and EMT related transcript factor snail, twist, zeb1, slug in OE-MFN1 97H cells and sh-MFN1 G2 cells. N-cadherin, vimentin, snail, twist, zeb1 and slug expression decreased in OE-MFN1 97H cells and increased in sh-MFN1 HepG2, while E-cadherin expression increased in OE-MFN1 97H cells and decreased in sh-MFN1 HepG2 cells. **(b, c)** Work flow of subcutaneous xenograft tumour model and orthotopic implantation tumour model. **(d, e)** Number of spontaneous lung metastasis in orthotopic xenograft models based on OE-MFN1 97H cells and sh-MFN1 G2 cells. MFN1 overexpression increased the number of lung metastasis, while sh-MFN1 G2 cells showed decreasing number of lung metastasis, though not statistically significant.

**
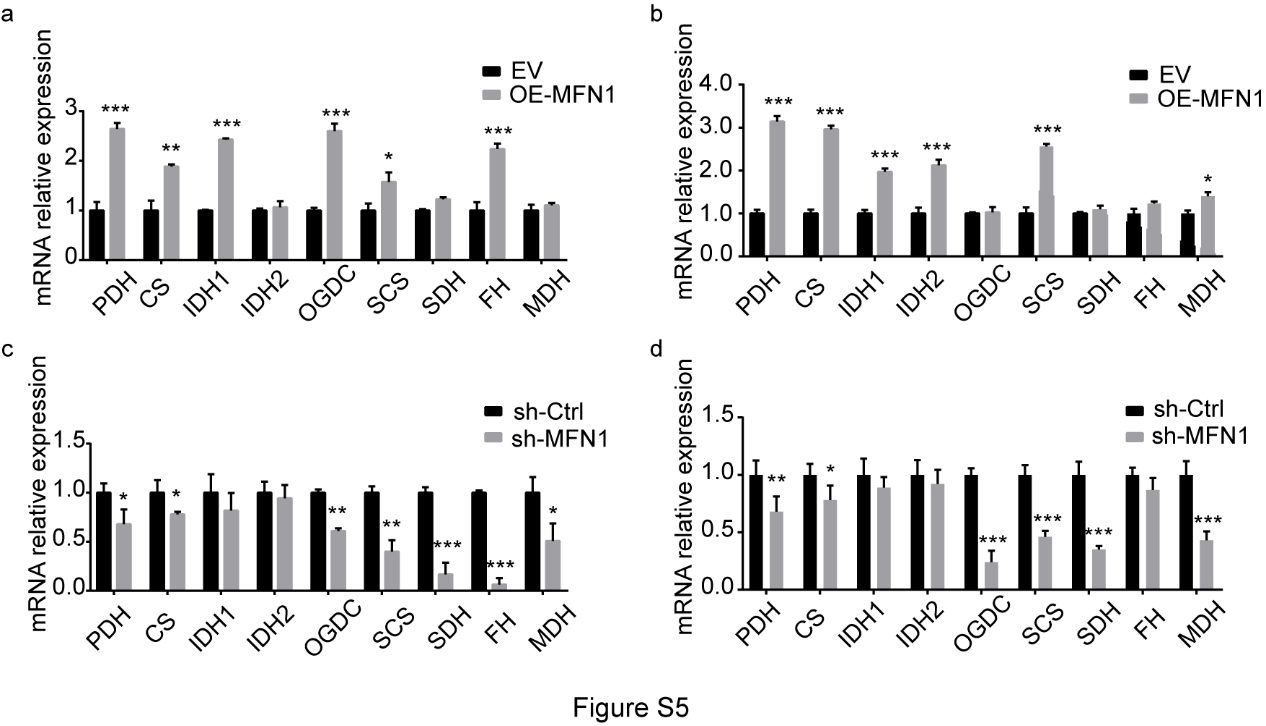
**

Fig. S5 **(a-d)** oxidative phosphorylation related enzymes were detected using qRT-PCR in OE-MFN1 97 cells, sh-MFN1 HepG2 cells and the xenograft tumours. Most enzymes involved in oxidative phosphorylation were upregulated in OE-MFN1 97H cells **(a)** and their xenograft tumour **(b)** and downregulated in sh-MFN1 G2 cells **(c)** and the xenografts **(d)**. N=6, Mean ± SEM, ***, P<0.001; **, P<0.01; *, P<0.05

**
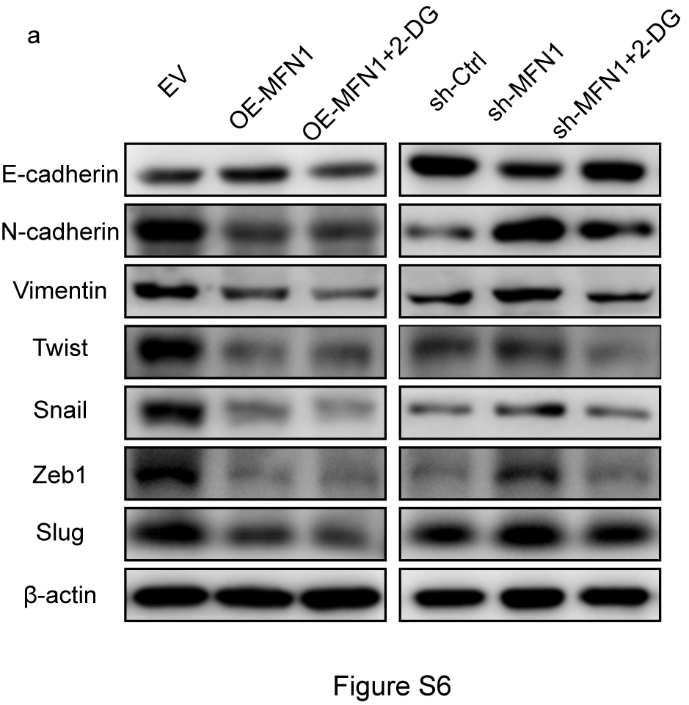
**

Fig. S6 **(a)** Expression of EMT related proteins were detected in 2-DG treated OE-MFN1 97H cells and sh-MFN1 HepG2 cells. When N-cadherin, vimentin, snail, twist, zeb1 and slug increased and E cadherin decreased in shMFN-1 HepG2 cells, 2-DG treated sh-MFN-1 HepG2 lost the changed expression. However, 2-DG did not change the expression level of EMT related protein of OE-MFN1 97H cells.

Supplementary Table1. TMA clinical information.


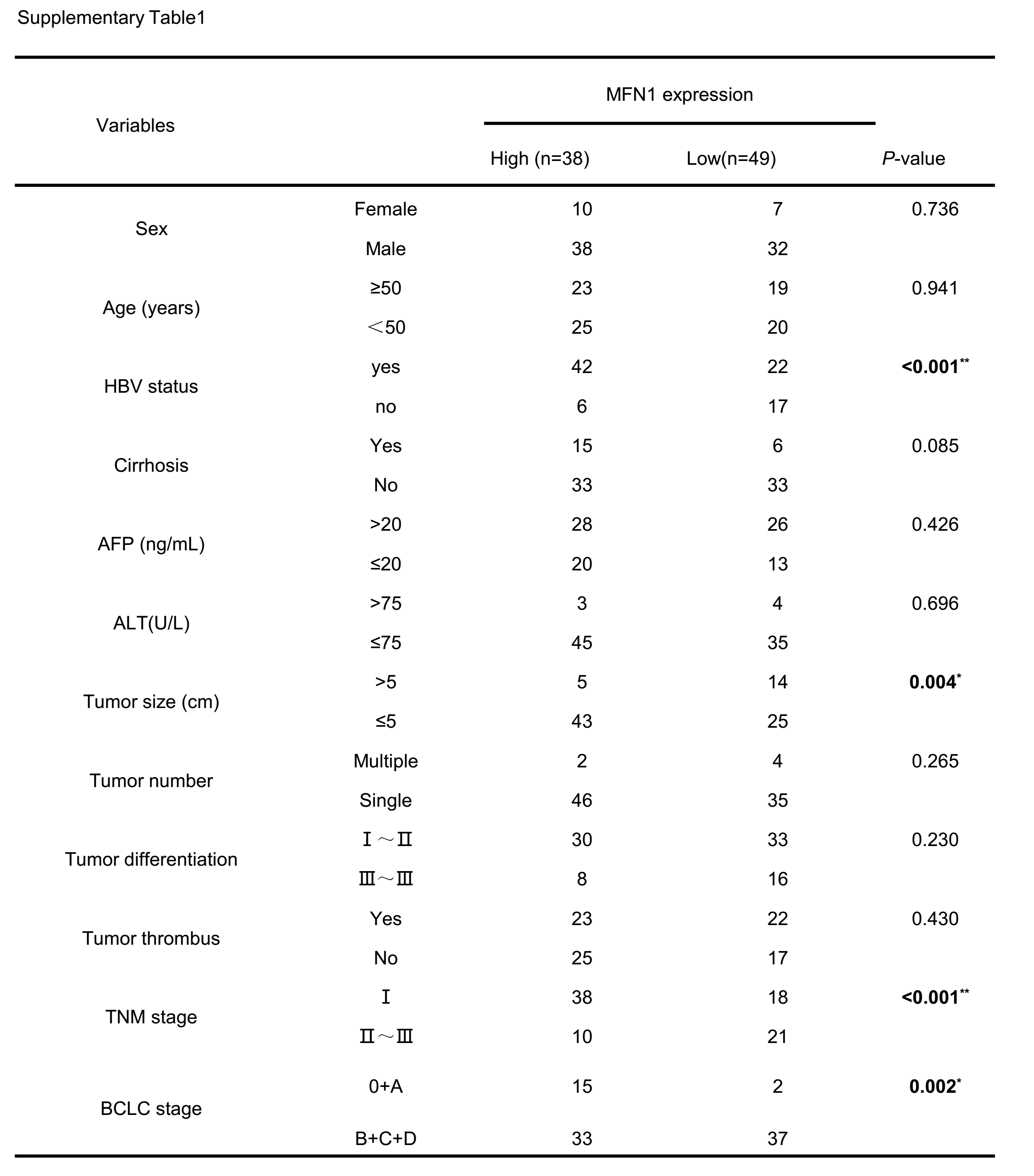


Supplementary Table2. Primer sequences used in the study.

| Primer name | | Sequence | |
| --- | --- | --- | --- |
|  | Forward | | Reverse |
| **Primer for qPCR** | | | |
| MFN1 | TGGCTAAGAAGGCGATTACTGC | | TCTCCGAGATAGCACCTCACC |
| MFN2 | CTCTCGATGCAACTCTATCGTC | | TCCTGTACGTGTCTTCAAGGAA |
| OPA1 | TGTGAGGTCTGCCAGTCTTTA | | TGTCCTTAATTGGGGTCGTTG |
| DNM1L | CTGCCTCAAATCGTCGTAGTG | | GAGGTCTCCGGGTGACAATTC |
| MFF | ACTGAAGGCATTAGTCAGCGA | | TCCTGCTACAACAATCCTCTCC |
| GLUT | GGCCAAGAGTGTGCTAAAGAA | | ACAGCGTTGATGCCAGACAG |
| HK2 | GAGCCACCACTCACCCTACT | | CCAGGCATTCGGCAATGTG |
| GPI | CAAGGACCGCTTCAACCACTT | | CCAGGATGGGTGTGTTTGACC |
| PFKFB2 | TGGGCCTCCTACATGACCAA | | CAGTTGAGGTAGCGTGTTAGTTT |
| ALDOA | ATGCCCTACCAATATCCAGCA | | GCTCCCAGTGGACTCATCTG |
| GAPDH | GGAGCGAGATCCCTCCAAAAT | | GCTCATAAGGACTACCGACTTGG |
| PGAM2 | AGAAGCACCCCTACTACAACTC | | TCTGGGGAACAATCTCCTCGT |
| ENO2 | AGCCTCTACGGGCATCTATGA | | TTCTCAGTCCCATCCAACTCC |
| PKM1/2 | ATGTCGAAGCCCCATAGTGAA | | TGGGTGGTGAATCAATGTCCA |
| LDH1 | ATGGCAACTCTAAAGGATCAGC | | CCAACCCCAACAACTGTAATCT |
| LDH2 | TGGTATGGCGTGTGCTATCAG | | TTGGCGGTCACAGAATAATCTTT |
| PDH | TGTGAACTGAGCAGGATCTATGG | | GGAATGTACGATGAGGAACAACA |
| CS | TGCTTCCTCCACGAATTTGAAA | | CCACCATACATCATGTCCACAG |
| IDH1 | TGTGGTAGAGATGCAAGGAGA | | TTGGTGACTTGGTCGTTGGTG |
| IDH2 | CGCCACTATGCCGACAAAAG | | ACTGCCAGATAATACGGGTCA |
| OGDC | GGCTTCCCAGACTGTTAAGAC | | GCAGAATAGCACCGAATCTGTTG |
| SCS | GTCCGCAGTCTTACGAGGAG | | GCTTGAGGGTCTGAATCTTGCT |
| SDH | AGCGCCTCAAGAATGAAGGTG | | GGGGAATGTAGACCCAACCC |
| FH | GGAGGTGTGACAGAACGCAT | | CATCTGCTGCCTTCATTATTGC |
| MDH | TCGGCCCAGAACAATGCTAAA | | GCGGCTTTGGTCTCGATGT |
| **Primer for sh-MFN1 RNA** | | | |
| Sh1 | CCGGGCGTTTAAGCAGCAGTTTGTACTCGAGTACAAACTGCTGCTTAAACGCTTTTTG | | AATTCAAAAAGCGTTTAAGCAGCAGTTTGTACTCGAGTACAAACTGCTGCTTAAACGC |
| Sh2 | CCGGTACGGAGCTCTGTACCTTTATCTCGAGATAAAGGTACAGAGCTCCGTATTTTTG | | AATTCAAAAATACGGAGCTCTGTACCTTTATCTCGAGATAAAGGTACAGAGCTCCGTA |
| Sh3 | CCGGGCCTTGTCTTAGCATTAGTTTCTCGAGAAACTAATGCTAAGACAAGGCTTTTTG | | AATTCAAAAAGCCTTGTCTTAGCATTAGTTTCTCGAGAAACTAATGCTAAGACAAGGC |
| **Primer for OE-MFN1 RNA** | | | |
|  | CCGGAATTCATGGCAGAACCTGTTTCTCCACT | | CGCGGATCCTTAGGATTCTTCATTGCTTGAAGGTA |
